# Interleukin-6 Downregulates the Expression of Vascular Endothelial-Cadherin and Increases Permeability in Renal Glomerular Endothelial Cells via the Trans-Signaling Pathway

**DOI:** 10.1007/s10753-022-01711-3

**Published:** 2022-07-23

**Authors:** Yong-Chang Yang, Hui Fu, Bo Zhang, Yu-Bin Wu

**Affiliations:** 1 Department of Pediatrics, Shengjing Hospital of China Medical University, No. 36 Sanhao Street, Heping District, Shenyang, 110004 Liaoning province China; 2 Department of Pediatrics, Tangshan Maternal and Child Health Hospital, Tangshan, 063000 Hebei province China

**Keywords:** interleukin 6, IgA nephropathy, vascular endothelial-cadherin, glomerular endothelial cells

## Abstract

The pathogenesis of IgA nephropathy (IgAN) is still unknown, but reportedly, interleukin 6 (IL-6) is involved in this process. However, its role in damaging glomerular endothelial cells is still unclear. Therefore, in this study, to clarify the mechanism of the pathogenesis of IgAN, we investigated the effect of IL-6 on the permeability of glomerular endothelial cells. A rat model of IgAN was established, and the animals divided into two groups, namely, the normal and IgAN groups. Glomerular endothelial cell injury was evaluated via electron microscopy. Furthermore, IL-6-induced changes in the permeability of human renal glomerular endothelial cells (HRGECs) were measured via trans-endothelial resistance (TEER) measurements and fluorescein isothiocyanate-dextran fluorescence. Furthermore, vascular endothelial-cadherin (VE-cadherin) was overexpressed to clarify the effect of IL-6 on HRGEC permeability, and to determine the pathway by which it acts. The classical signaling pathway was blocked by silencing IL-6R and the trans-signaling pathway was blocked by sgp30Fc. In IgAN rats, electron microscopy showed glomerular endothelial cell damage and western blotting revealed a significant increase in IL-6 expression, while VE-cadherin expression decreased significantly in the renal tissues. IL-6/IL-6R stimulation also significantly increased the permeability of HRGECs (*p* < 0.05). This effect was significantly reduced by VE-cadherin overexpression (*p* < 0.01). After IL-6R was silenced, IL-6/IL-6R still significantly reduced VE-cadherin expression and sgp30Fc blocked the trans-signaling pathway as well as the upregulation of IL-6/IL-6R-induced VE-cadherin expression. This suggests that IL-6 mainly acts via the trans-signaling pathway. IL-6 increased the permeability of HRGECs by decreasing the expression of VE-cadherin via the trans-signaling pathway.

**Supplementary Information:**

The online version contains supplementary material available at 10.1007/s10753-022-01711-3.

## Background

IgA nephropathy (IgAN) is a chronic kidney disease that eventually leads to end-stage renal disease. Even though it is the most common primary glomerular disease, its pathogenesis is still unclear [1]. Furthermore, it has been reported that damage to the glomerular endothelial cells is closely associated with IgAN progression [2]. However, previous studies on glomerular diseases have been primarily focused on mesangial cells and podocytes, although endothelial cell injury is also involved in the development of several glomerular diseases [3].

A semi-permeable membrane barrier is formed between endothelial cells via adherens junctions (AJs), tight junctions, and a series of adherent molecules. These include vascular endothelial-cadherin (VE-cadherin), which is a key transmembrane protein that is involved in endothelial adhesion and is expressed only in endothelial cells [4]. The disruption of AJs reduces VE-cadherin expression and increases endothelial permeability [5]. Furthermore, VE-cadherin on the cell surface interacts with β-catenin through its cytoplasmic tail [6]. It has been observed that β-catenin phosphorylation leads to the disassociation of VE-cadherin [7], and the dissociated VE-cadherin is degraded by endocytosis. Furthermore, VE-cadherin expression is significantly reduced in ischemia–reperfusion renal injury [8]. However, the changes in its expression level in IgAN have not yet been studied.

Inflammatory cytokine, interleukin 6 (IL-6), which is involved in the development of several diseases [9], is known to play a role in IgAN pathogenesis. Its concentration in the urine of patients with IgAN is associated with renal function [10] and can be used to predict long-term renal function outcomes in such patients [11]. The development of IgAN is often secondary to mucosal infection, which leads to elevated IL-6 levels locally or throughout the body [12]. Additionally, IL-6 can combine with the IL-6R/gp130 complex to activate the JAK/STAT3 pathway, which induces STAT3 expression in the nucleus and increases the production of galactose-deficient IgA1 (Gd-IgA1) [13]. Taken together, this evidence suggests that IL-6 plays an important role in IgAN pathogenesis. However, whether it is associated with increased glomerular endothelial permeability in IgAN or exerts an effect on VE-cadherin expression remains to be determined. Thus, using an IgAN rat model and human renal glomerular endothelial cells (HRGECs), we aimed in this study to determine whether IL-6 can cause decreased VE-cadherin expression and increased renal glomerular endothelial cell permeability.

## Methods

### Ethical Approval

This study was approved by the Ethics Committee of Shengjing Hospital of China Medical University (No. 2017PS13K), and all the procedures performed in the animal experiments were in accordance with the ethical standards of this institution.

### Animal Model

Twenty specific pathogen-free (SPF) 6-week-old male Sprague–Dawley rats, weighing 180 ± 10 g, were purchased from the Beijing Huafukang Bioscience Corporation. Thereafter, the animals were acclimatized for 7 days in the SPF-class animal room of Shengjing Hospital before experimentation and were given access to sufficient amounts of food and water. At the end of this 7-day period, all the rats were active, had shiny coats, and tested negative in two qualitative tests for urinary proteins. Next, they were randomly divided into two groups, namely, the normal group (*n* = 10) and the IgAN group (*n* = 10). To establish the IgAN rat model, bovine serum albumin (BSA; Sigma, St. Louis, MO, USA), carbon tetrachloride (CCl_4_; Sinopharm Chemical Reagent Co. Ltd., Shanghai, China), and lipopolysaccharide (LPS; Sigma, St. Louis, MO, USA), were used as previously described [14]. Briefly, the IgAN model rats were intragastrically administered 400 mg/kg BSA once every 2 days and subcutaneously injected 0.1 mL CCl_4_ dissolved in 0.4 mL castor oil weekly for 8 weeks. From the fifth week onwards, 0.05 mg of LPS was injected once a week through the caudal vein for 4 weeks. Simultaneously, the rats in the normal group were administered the same volume of distilled water intragastrically, and the same volume of sodium chloride solution was injected subcutaneously.

At the end of the eighth week, all the rats were anesthetized using pentobarbital sodium and sacrificed via the dislocation of the cervical spine. Abdominal aortic blood (3 mL) was then collected and centrifuged at 1000 × *g* for 10 min within 30 min. A portion of the kidney tissue was fixed with 4% paraformaldehyde and embedded in paraffin, while the remaining kidney tissue sections were frozen in liquid nitrogen in preparation for subsequent immunofluorescence (IF) and western blotting (WB) procedures.

### Biochemical Index Assessment

At weeks 1, 3, 6, and 8, 24-h urine samples were collected using the metabolic cage method under fasting and free access to drinking water conditions, and urinary red blood cell (RBC) counts and protein levels were determined. Specifically, urinary RBCs were counted in 10 fields at high magnification (× 400) using a red blood cell counting plate. Samples with the presence of three RBCs per field were considered positive for hematuria. Regarding the detection of protein levels, the urine samples were centrifuged at 3500 × *g* for 5 min, and the supernatant obtained was analyzed using the Coomassie Blue method.

### HRGEC Culture

HRGECs were purchased from BeNa Culture Collection (Beijing, China) and grown in 90% DMEM-H (Sigma, St. Louis, MO, USA) and 10% fetal bovine serum (FBS, Sigma) at 37 °C with 5% CO_2_. Only the first five generations of the HRGECs were used.

### Direct IF

IgA deposition on the mesangial tissue of rats in each group was observed via IF. In brief, frozen renal tissue samples were washed with phosphate buffered saline (PBS) and fixed with cold acetone at 4 °C for 30 min. Thereafter, the tissue sections were incubated with fluorescein isothiocyanate (FITC)-labeled goat anti-rat IgA antibody (1:100, Abcam, UK) for 1 h at 27 °C. After washing with PBS, the sections were sealed with a fluorescent anti-quenching agent and observed under a Nikon fluorescence microscope (Nikon, Tokyo, Japan).

### Electron Microscopy

To perform electron microscopy experiments, small tissue samples were fixed in 2% glutaraldehyde, washed with PBS, and further fixed with 1% osmium tetroxide. Thereafter, the specimens were dehydrated using a series of graded methanol solutions and embedded in an EMBed 812 epoxy resin. Next, ultrathin slices (60 nm) were cut out using an RMC PowerTome and stained with uranyl acetate and lead citrate. Finally, the slices were observed under a Hitachi H-7600 transmission electron microscope, and images were captured using an AMT 1 K digital imaging camera.

### WB

WB analysis was performed as described previously [14]. The primary antibodies used for this analysis included VE-cadherin (sc-9989, 1:500, Santa Cruz Biotechnology, Santa Cruz, CA, USA), IL-6 (sc-32296, 1:500, Santa Cruz Biotechnology), β-actin (WL01372, 1:500, Wanleibio, Shenyang, China), and glyceraldehyde phosphate dehydrogenase (GAPDH) (TA309157, 1:1000; Zhongshan Jinqiao, Beijing, China).

### PCR

Total RNA from cultured cells was isolated using TRIzol reagent (Invitrogen, Carlsbad, CA, USA) following the manufacturer’s protocol. Thereafter, 100 ng of the total RNA was reverse transcribed to cDNA using a Takara reverse transcription kit (Dalian, China) and amplified via qRT-PCR using TB Green Premix Ex Taq II (Takara, Dalian, China). β-Actin was used as an internal control. The primer sequences used are presented in Supplementary Table S1.

### Lentivirus Infection

Stable cell lines overexpressing the VE-cadherin gene or with IL-6R silenced were established via lentivirus transfection. Lentiviral vectors packaged with the full-length VE-cadherin gene for VE-cadherin overexpression or shRNA for IL-6R silencing were provided by Fenghui Company (Changsha, China). Briefly, the VE-cadherin overexpressing lentivirus (Lv-VEcad) or the IL-6R silencing lentivirus (sh-IL-6R) and their corresponding negative controls (Lv-NC) were introduced into HRGEC cells, and thereafter, cell lines with stable expression were screened.

### Pathological Evaluation and Immunofluorescence Staining

Paraffin-embedded kidney Sects. (3-μM thick) were prepared. Next, to analyze the proliferation of glomerular cells, the sections were stained with periodic acid-Schiff (PAS) (Beyotime, Shanghai, China) according to the manufacturer’s instructions. HRGECs cultured on Lab-Tek chamber slides were fixed with paraformaldehyde and rinsed in PBS. Thereafter, the cell membranes were permeabilized using 0.5% Triton-100 for 30 min and sealed with 1% BSA at 27 ℃ for 1 h. Immunohistochemical staining was then performed followed by incubation of the cell slides with a monoclonal antibody specific for VE-cadherin (sc-9989, 1:50, Santa Cruz Biotechnology) overnight at 4 °C. In the next step, the cell slides were incubated with FITC-conjugated secondary antibodies (ab6717, 1:200, Abcam, Cambridge, UK) at 27 ℃ in the dark for 1 h, after which they were stained with 4′, 6-diamidino-2-phenylindole (DAPI; Beyotime, Shanghai, China) for 5 min. Fluorescent images were then captured using a confocal microscope (BX53, Olympus, Japan). All the biopsy results were evaluated in an investigator-blinded manner.

### Cytotoxicity Assay

The cytotoxicity of IL-6 and IL-6/IL-6R was assessed using the Cell Counting Kit-8 (CCK-8). Specifically, cells were grown in 96-well plates (3000 cells/well) and cultured in serum-free medium for 12 h followed by the addition of IL-6 or IL-6/IL-6R at the indicated concentrations. Subsequently, the cells were grown over specified periods (24 and 48 h), and finally, 10 μL of [2-(2-methoxy-4-nitrophenyl)-3-(4-nitrophenyl)-5-(2,4-disulfophenyl)-2H-tetrazolium monosodium salt was added, and the absorbance of each well was measured at 450 nm after incubation at 37 °C for 1 h. Five independent replicates were used for each time point.

### Transendothelial Electrical Resistance (TEER) Assay

A 4 × 10^4^ cells/mL, suspension of HRGECs was prepared using complete medium containing 90% DMEM-H and 10% FBS. Next, 500 µL of the cell suspension was added to the upper chamber of a 3-µm diameter polycarbonate 12-well Transwell plate (area 1.12 cm^2^), while the complete medium (1.5 mL) was added to the lower chamber. For vacuum comparison, the same amount of medium was added to uninoculated cell holes and continuous culturing was performed for 14 days, with the medium changed every 48 h. TEER = (sample reading from inoculated cells–sample reading from blank contrast) × 1.12 was measured daily, and given that the TEER value became stable following culturing for 14 days, we considered that a monolayer cell model had been established. Thus, 14 days of intervention was employed in subsequent follow-up experiments.

The stimulants were grouped as follows: control group, drug solvent containing 90% DMEM-H and 10% FBS medium (negative control); IL-6 group, IL-6 stimulation only; IL-6/IL-6R group, IL-6 combined with 15-fold IL-6R stimulation as previously described [15]. Three repeated holes were set up for each group, and the changes in cell resistivity were monitored for 24 h and 48 h. All the experiments were carried out in 5% CO_2_ at 37 °C.

### Transendothelial Permeability Assay

To perform the transendothelial permeability assay, 500-µL HRGEC suspensions containing 2 × 10^4^ cells/well were added to the upper compartment of the Transwell co-culture plate, while 500 µL of complete medium was added to the lower compartment. Thereafter, the cultures were allowed to grow to confluence on the Transwell permeable supports for 14 days, after which the HRGEC monolayers obtained were treated with different reagents for 24 h and 48 h with the control, IL-6, and IL-6/IL-6R treatments. Next, all the culture media in the lower chamber were removed and replaced with fresh medium. Then, in the upper chamber, 0.5 mL of medium containing fluorescein isothiocyanate-dextran (FITC-dextran, 0.05 mg/mL) was added followed by culturing in the dark for 30 min. Finally, the amount of FITC-dextran that diffused into the bottom chamber was determined using a fluorescence microplate reader.

### Enzyme-Linked Immunosorbent Assay (ELISA) for IL-6

Serum IL-6 levels were detected using the Rat IL-6 Quantikine ELISA Kit (R6000B, R&D Systems, Minneapolis, MN, USA) according to the manufacturer’s instructions.

### Statistical Analysis

All statistical analyses were performed using SPSS software version 26.0 (IBM, New York, NY, USA). Results corresponding to continuous variables are expressed as mean ± standard deviation (SD). Comparisons between two groups were performed using the *t*-test, while comparisons between three groups were performed using one-way analysis of variance (ANOVA). If the ANOVA showed significant differences, Tukey’s multiple comparisons were further performed to determine which groups differed. Statistical significance was set at *p* < 0.05.

## Results

### Successful Establishment of the IgAN Rat Model

**Fig. 1:**
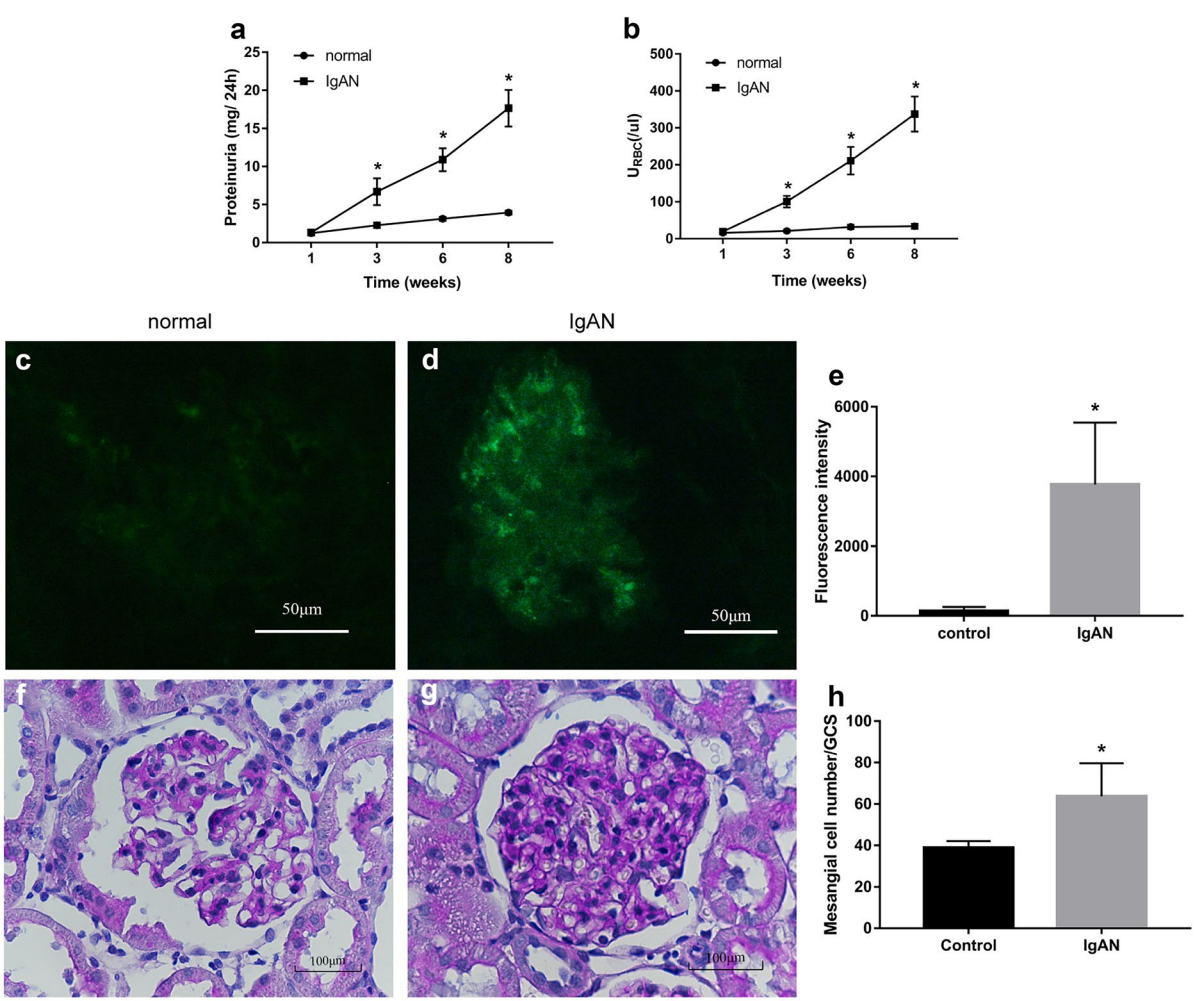
Hematuria, proteinuria, and IgA deposition in rats with IgAN nephropathy. From third week, rats in the IgAN group showed significantly higher (**a**) proteinuria and (**b**) hematuria than the normal group. In direct immunofluorescence, green fluorescence in the glomerulus represents IgA deposition. At high magnification (200×), (**c**) rats in the normal group showed faintly visible IgA deposition in their glomeruli, while (**d**) rats in the IgAN group showed a high amount of dazzling IgA deposition in their glomeruli. (**e**) Fluorescence intensity corresponding to the IgAN group significantly higher than that corresponding to the normal group (*p* < 0.01). PAS staining in paraffin sections showed (**f**) no proliferation of mesangial cells in the normal group, and (**g**) obvious proliferation of mesangial cells in the IgAN group. (**h**) Statistics showing significantly higher mesangial cell proliferation in the IgAN group than in the normal group (*p* < 0.01). These findings presented as the mean ± SD were analyzed by performing the *t*-test (*n* = 6). *p < 0.01, IgAN vs. normal group. IgAN, IgA nephropathy; URBC, urine red blood cells; SD, standard deviation

In the IgAN model group, hematuria and 24-h proteinuria continued to increase during weeks 3, 6, and 8, and the difference was statistically significant compared with the control group (*p* < 0.01, Fig. 1a, b).

For the normal group, at high magnification (× 200), IgA direct IF staining showed only a few seemingly visible IgA deposits in the glomeruli (Fig. 1c). However, at the same high magnification (× 200), the glomeruli of rats in the IgAN group showed a high amount of dazzling granular green fluorescence deposition (Fig. 1d). Thus, the fluorescence intensity corresponding to the IgAN group was significantly higher than that corresponding to the normal group (*p* < 0.01, Fig. 1e), indicating that the IgAN model was successfully established. PAS staining revealed no mesangial cell proliferation in renal tissue from rats in the normal group (Fig. 1f). However, rats in the IgAN model group showed mild to moderate mesangial cell and mesangial matrix proliferation (Fig. 1g), and compared with normal group, the number of mesangial cells corresponding to the IgAN group was significantly increased (*p* < 0.01, Fig. 1h).

### Injury to Glomerular Endothelial Cells in IgAN Rats

**Fig. 2:**
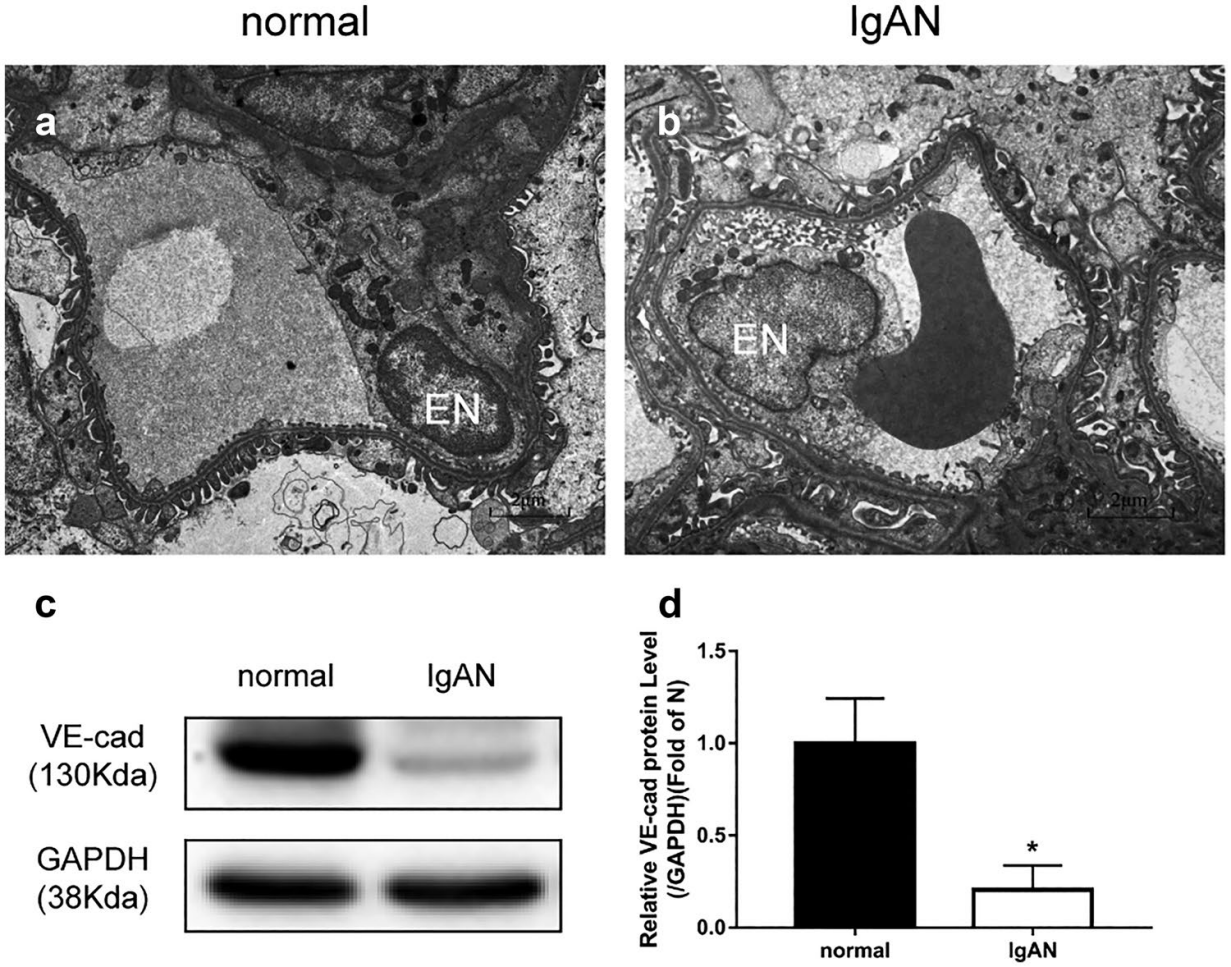
Glomerular endothelial cell injury in IgAN rats. Electron microscopy showed that **a** the glomerular endothelial cells were closely attached to the basement membrane for rats in the normal group (*n* = 6), but **b** were separated from the basement membrane for rats in the IgAN group (*n* = 4). **c**, **d** IgAN group showing significantly lower VE-cadherin expression than normal group based on western blot analysis (*p* < 0.01). *p < 0.01, IgAN vs. normal group. IgAN, IgA nephropathy; EN, endothelial cells; VE-cad, VE-cadherin; GAPDH, glyceraldehyde phosphate dehydrogenase.

Electron microscopy showed that the glomerular endothelial cells were closely attached to the basement membrane in renal tissues in rats in the normal group (Fig. 2a), while those in rats from the IgAN group showed glomerular endothelial cells that were vacuolated and separated from the basement membrane (Fig. 2b).

Additionally, WB showed that the level of VE-cadherin protein corresponding to the IgAN group was significantly reduced compared with that corresponding to the normal group (*p* < 0.01, Fig. 2c, d).

### IL-6 Changes in IgAN Rats

**Fig. 3:**
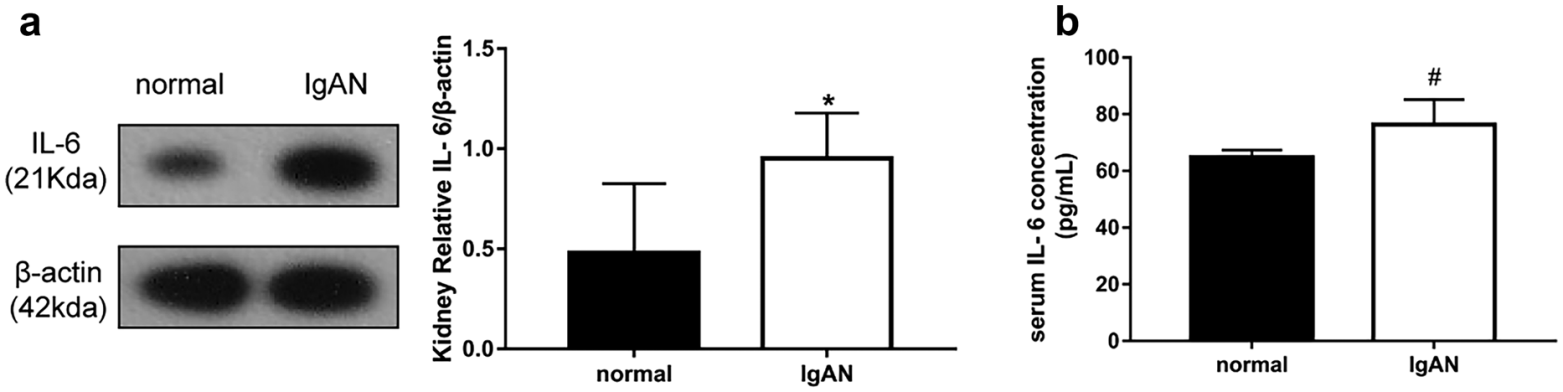
Changes in IL-6 level. **a** IgAN group showing a significantly higher renal IL-6 level than the normal group based on western blot analysis (*n* = 7), **b** IgAN group showing a significantly higher serum IL-6 level than the normal group based on ELISA (*n* = 6). *p < 0.01, compared with the normal group, ^#^*p* < 0.05, compared with the normal group. IgAN, IgA nephropathy; IL-6, interleukin 6.

The IgAN group showed a notable increase in kidney IL-6 levels based on the WB (*p* < 0.01, Fig. 3a). Furthermore, the observed changes in serum IL-6 levels, based on ELISA, were consistent with these WB results (*p* < 0.05, Fig. 3b).

### IL-6 Increases HRGEC Permeability

**Fig. 4:**
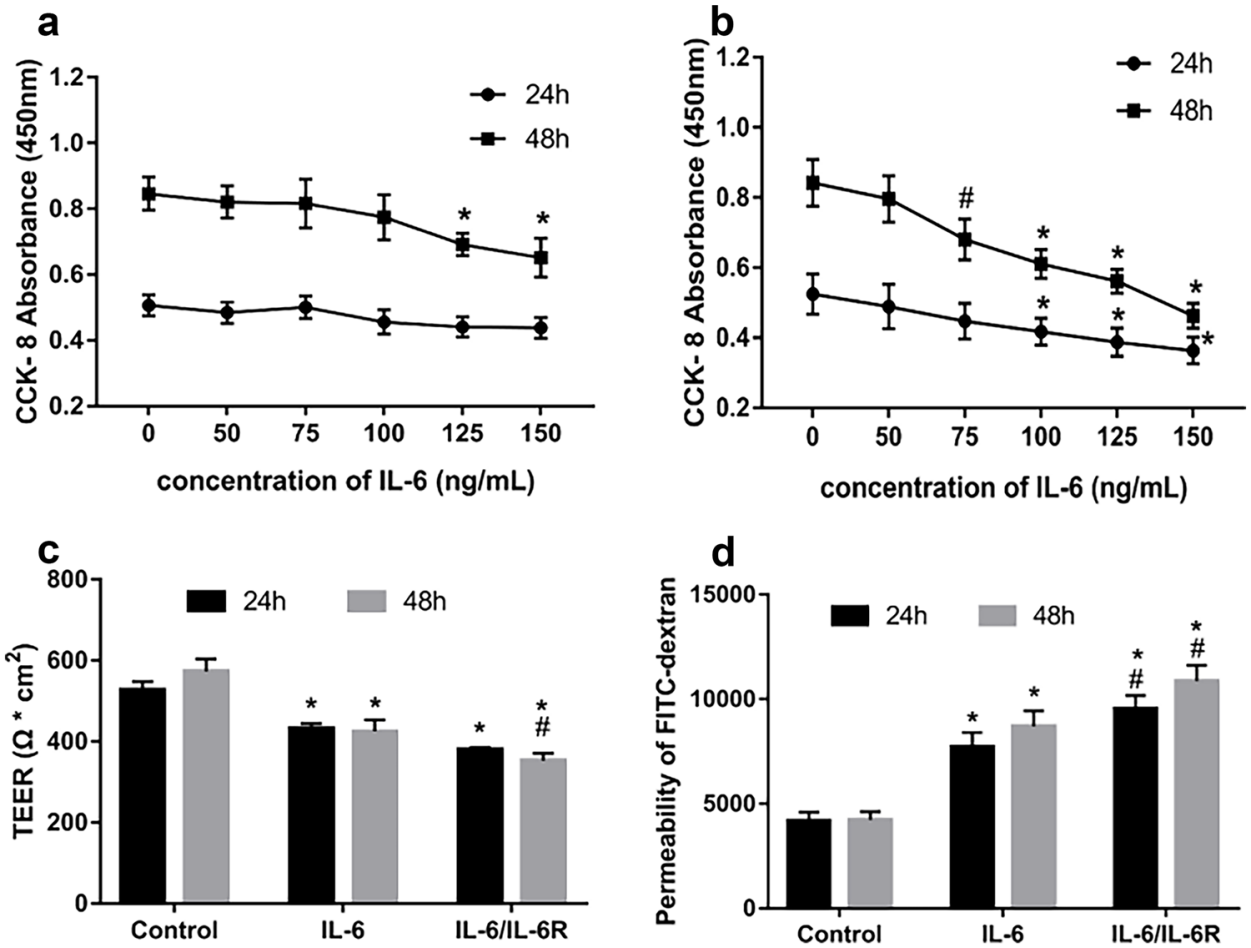
Effects of IL-6 on HRGEC cell viability and permeability. **a** HRGEC cell viability was assessed via CCK-8 assay by exposing them to various concentrations of IL-6 (0, 50, 75, 100, 125, and 150 ng/mL) for 24 and 48 h. Cell proliferation was inhibited only at 48 h when the IL-6 concentration was greater than or equal to 125 ng/mL (*p* < 0.01, *n* = 5, *p < 0.01, compared with the control treatment). **b** HRGECs were exposed to various concentrations of IL-6 and IL-6R (IL-6: IL-6R = 1:15) for 24 and 48 h. At 24 h of stimulation, cell proliferation was inhibited when the IL-6 concentration was greater than or equal to 125 ng/mL (*p* < 0.01). At 48 h of stimulation, cell proliferation was inhibited when the IL-6 concentration was greater than or equal to 75 ng/mL (*p* < 0.05, *n* = 5, *p < 0.01, compared with the 0 ng/mL treatment; ^#^*p* < 0.05, compared with control treatment). **c** Statistical analysis showing that IL-6 or IL-6/IL-6R significantly reduced TEER values at 24 and 48 h (*p* < 0.01). At 48 h, the TEER value corresponding to the IL-6/IL-6R group decreased to a greater extent than that corresponding to the IL-6 group (*n* = 3, *p < 0.01, compared with control; ^#^*p* < 0.05, compared with IL-6 treatment). **d** FITC-dextran cell permeability analysis showed that IL-6 or IL-6/IL-6R increased HRGEC permeability at 24 and 48 h, and the IL-6 /IL-6R group induced a more significant increase in permeability both at 24 and 48 h (*n* = 3, *p < 0.01, compared with the control treatment; #*p* < 0.05, compared with the IL-6 treatment). Data are presented as mean ± SD. HRGEC, human renal glomerular endothelial cell; IL-6, interleukin-6; IL-6R, interleukin-6 receptor; CCK-8, Cell Counting Kit-8; TEER, transendothelial electrical resistance; SD, standard deviation.

To determine the effect of IL-6 concentration on HRGEC permeability, we first conducted a CCK-8 assay to clarify the effect of IL-6 on the proliferation of HRGECs. As it is unclear whether IL-6 works through the classical or trans-signaling pathway, we designed the IL-6-only treatment group (0, 50, 75, 100, 125, 150 ng/mL), and the IL-6 combined with IL-6 receptor (IL-6R) treatment group, with a 15-fold higher IL-6 concentration relative to the IL-6-only treatment, as previously described [15]. This allowed us to stimulate HRGECs for 24 and 48 h, to the end of detection of cell proliferation. The results thus obtained showed that the inhibition of HRGEC proliferation was lower following the IL-6-only treatment. However, when IL-6 at concentrations greater than or equal to 125 ng/mL was used based on 48-h stimulation, HRGEC proliferation was significantly inhibited, compared with the outcome of the control treatment (*p* < 0.01, Fig. 4a). Furthermore, when IL-6 was combined with 15-fold IL-6R, the concentration of IL-6 was greater than or equal to 75 ng/mL, and cell proliferation was also significantly inhibited compared with the control treatment (*p* < 0.05, Fig. 4b). These results indicated that the IL-6-only treatment had a weak inhibitory effect on the proliferation of HRGECs, and possibly, primarily inhibited the proliferation of HRGECs via the trans-signaling pathway involving soluble IL-6R.

Based on preliminary experiments, 100 ng/mL IL-6 and 1500 ng/mL IL-6R were selected to stimulate HRGECs. Thus, three treatment groups, namely, the normal, IL-6, and IL-6/IL-6R groups, were considered to investigate the effect of IL-6 on HRGEC permeability. We first detected TEER and showed that IL-6 or IL-6/IL-6R stimulation significantly reduced TEER at 24 h and 48 h (*p* < 0.01) compared with the control treatment. Additionally, at 48 h, the IL-6 /IL-6R group displayed a greater decrease in the TEER value than the IL-6 group (*p* < 0.05) (Fig. 4c). Thus, we further applied FITC-dextran to test transendothelial cell permeability. We observed that IL-6 or IL-6/IL-6R stimulation significantly increased HRGEC permeability compared to the control treatment (*p* < 0.01), with the IL-6/IL-6R group showing a more significant increase (*p* < 0.05, Fig. 4d).

### IL-6 Affects the Permeability of HRGEC Cells Through VE-Cadherin

**Fig. 5:**
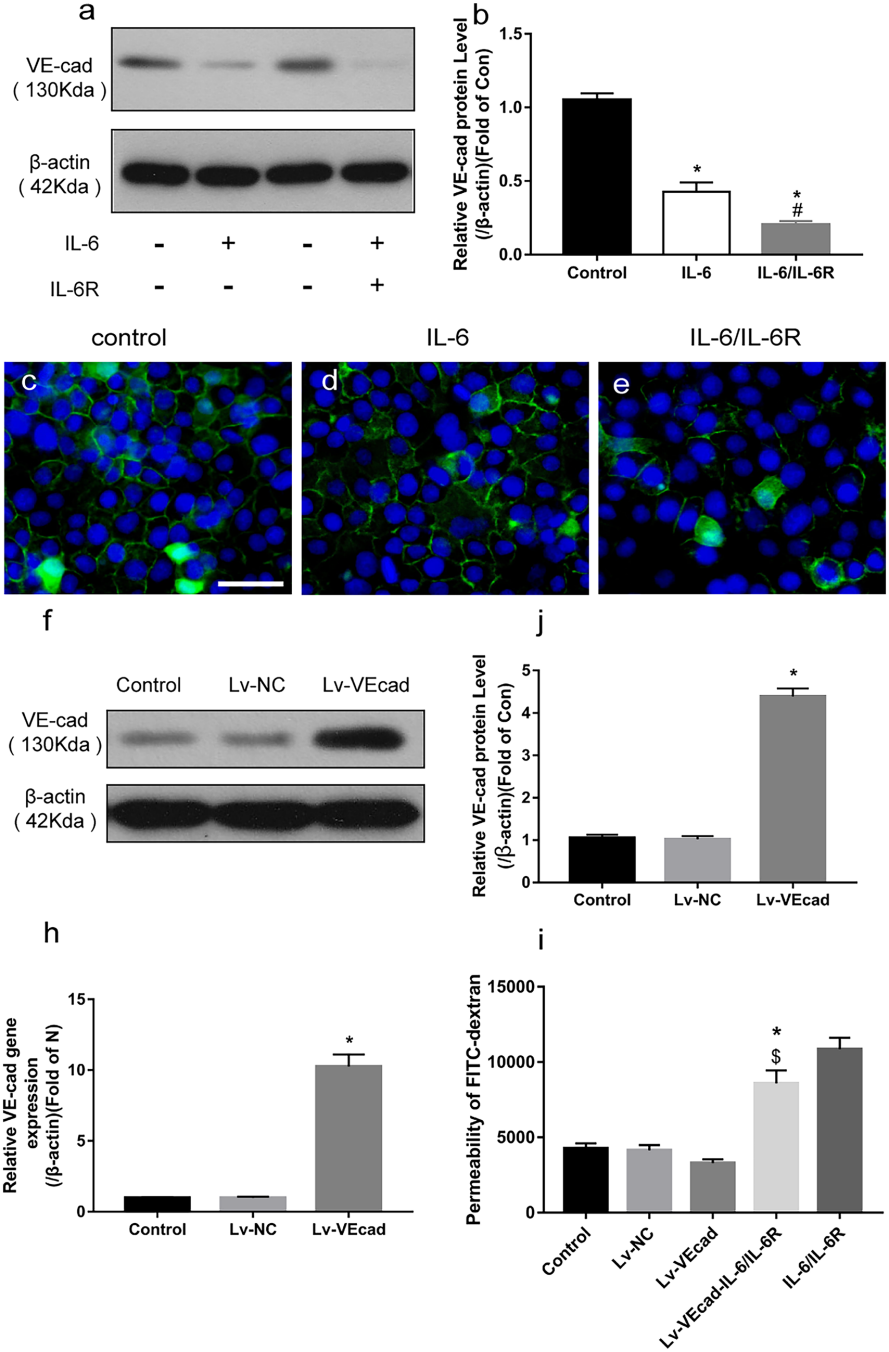
IL-6 increases cell permeability by downregulating VE-cadherin expression. **a**, **b** Western blot analysis results showing that IL-6 or IL-6/IL-6R significantly reduced VE-cad expression, with IL-6/IL-6R showing a stronger downregulating effect in this regard than the IL-6-only treatment (*p* < 0.01). **c**–**e** Green fluorescence represents VE-cadherin protein expression in different HRGEC treatment groups. **c** Control group showing continuously linear VE-cadherin expression in HRGECs. (**d**) Discontinuous linear decrease in VE-cadherin expression after IL-6 stimulation. **e** Almost absent intercellular VE-cadherin expression after IL-6/IL-6R stimulation. **f**–**h** Significantly increased VE-cadherin protein and mRNA levels after lentivirus transfection, indicating successful VE-cadherin over-expression. **i** Compared with the control group, the IL-6/IL-6R-stimulated permeability of the cells in the Lv-VEcad group was still increased, but significantly lower than that corresponding to the IL-6/IL-6R group, suggesting that VE-cadherin overexpression can block the increased IL-6/IL-6R-induced permeability (*n* = 3); *p < 0.01, compared with the control; ^#^*p* < 0.01, compared with the IL-6 group; ^$^*p* < 0.01, compared with IL-6/IL-6R group. VE-cad, VE-cadherin; HRGECs, human renal glomerular endothelial cells; IL-6, interleukin-6; IL-6R, interleukin-6 receptor; Lv-NC, lentivirus-transfected negative control group; Lv-VEcad, lentivirus-transfected VE-cadherin over-expression group

WB showed that IL-6 or IL-6/IL-6R significantly downregulated VE-cadherin protein expression in HRGECs (Fig. 5a). The IF staining image, with green linear fluorescence indicating VE-cadherin and blue fluorescence indicating nuclei, showed that IL-6/IL-6R stimulation significantly decreased green fluorescence in HRGECs (Fig. 5c–e).

To determine whether VE-cadherin was necessary for IL-6 to affect HRGEC permeability, we over-expressed VE-cadherin in HRGEC cells via lentivirus transfection. Thereafter, WB showed that the expression of VE-cadherin protein was significantly increased after transfection (Fig. 5f, j). Furthermore, PCR indicated that VE-cadherin mRNA expression was also significantly upregulated (Fig. 5h), indicating that the overexpression was successful. VE-cadherin overexpressing cells re-stimulated with IL-6/IL-6R showed inhibition of cell permeability compared with the normal cells (*p* < 0.01). This inhibition notwithstanding, these re-stimulated VE-cadherin overexpressing cells still showed significantly higher permeability than the VE-cadherin over-expression group without IL-6/IL-6R stimulation (*p* < 0.01, Fig. 5i). These results suggest that VE-cadherin is involved in the process of IL-6/IL-6R-induced increase in cell permeability.

### IL-6 Affects VE-Cadherin Expression in HRGECs via the Trans-Signaling Pathway

**Fig. 6:**
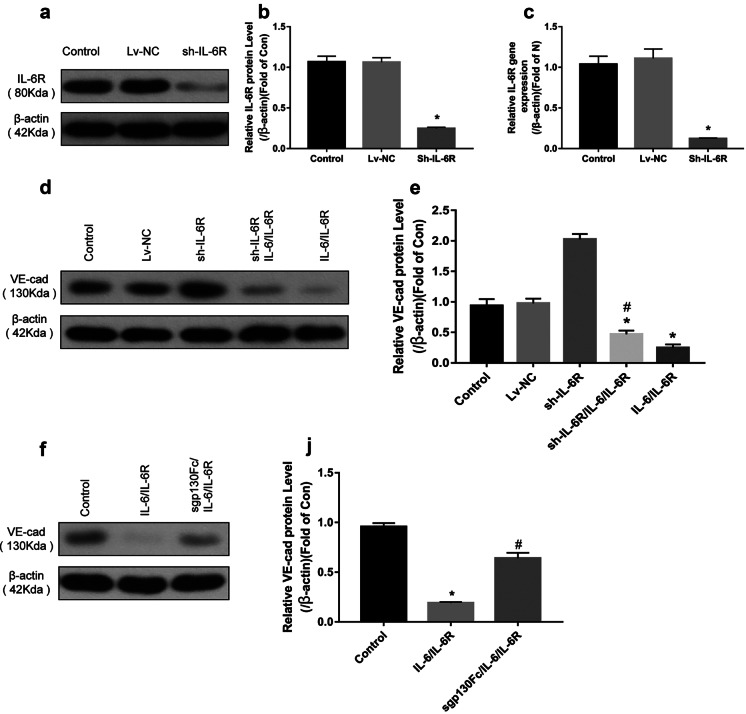
IL-6 affects VE-cadherin expression in HRGEC cells via trans-signaling pathway. **a**–**c** Significantly decreased IL-6R protein and mRNA expression levels after lentivirus transfection, indicating successful IL-6R silencing. **d** After IL-6R silencing, IL-6/IL-6R still showed the ability to downregulate VE-cadherin expression via the trans-signaling pathway. **e** The sh-IL-6R/IL-6/IL-6R group showing significantly lower VE-cadherin expression than the control group (*p* < 0.01), but higher than the IL-6 group (*p* < 0.01). **f** After the use of sgp130Fc to block the trans-signaling pathway, VE-cadherin expression significantly increased. **j** The sgp130Fc/IL-6/IL-6R group showing significantly higher VE-cadherin expression than the IL-6/IL-6R group (*p* < 0.01) (*n* = 3). *p < 0.01, compared with the control group; ^#^*p* < 0.01, compared with the IL-6/IL-6R group. IL-6, interleukin-6; IL-6R, interleukin-6 receptor; sh-IL-6R, IL-6R silenced; Lv-NC, negative control.

Previous studies have shown that gp130 is widely expressed in cells [16], and in this study, we also confirmed its expression in HRGECs via WB (Supplementary Fig. S2). Thus, we then silenced IL-6R expression via lentivirus transfection, and the results of WB and PCR further indicated that IL-6R protein and mRNA expression decreased significantly after silencing, indicating that the IL-6R silencing was successful (Fig. 6a–c). Next, we administered IL-6/IL-6R to stimulate HRGEC cells again, such that only the trans-signaling pathway could play a role. The results obtained showed that the expression of VE-cadherin in HRGEC cells did not increase (Fig. 6d, e), indicating that IL-6 primarily downregulated VE-cadherin expression via the trans-signaling pathway, rather than the classical pathway.

Additionally, sgp130Fc (671-GP, R&D, USA) specifically inhibits the IL-6 trans-signaling pathway by binding to the IL-6/IL-6R complex [17]. Thus, we applied sgp130Fc to block the trans-signaling pathway, and observed that VE-cadherin expression in HRGECs increased significantly (Fig. 6f, j). This observation further confirmed that IL-6 downregulated VE-cadherin expression in HRGEC cells primarily via the trans-signaling pathway.

## Discussion

IL-6 is involved in the occurrence and development of various diseases. Reportedly, it affects the physiological functions of podocytes [18], mesangial cells [19], endothelial cells [20], and tubular epithelial cells [21] during the progression of kidney diseases, such as IgAN [22], lupus nephritis [23], and diabetic nephropathy [24]. The incidence of Castleman disease is also closely related to IL-6 expression, and cases of IgAN combined with Castleman disease, wherein IL-6 antagonist treatment resolves IgAN, have also been reported [25]. Furthermore, clinical studies have shown that serum IL-6 levels in patients with IgAN are significantly elevated and are negatively correlated with the estimated glomerular filtration rate [26]. In this study, we also observed significantly increased IL-6 levels in sera and kidney tissue samples from rats in the IgAN group compared with the observations made for sera and kidney tissue samples from rats in the normal group, possibly suggesting the involvement of IL-6 in IgAN pathogenesis. The increased IL-6 level in renal tissue may be due to secondary infection and renal immune complex stimulation, as IgAN often occurs secondary to respiratory tract infection, which increases serum IL-6 levels. Furthermore, immune complex deposition in the kidneys is a direct cause of glomerular inflammation and mesangial hyperplasia [27]. Immune complex in the mesangial region can also stimulate mesangial cells to secrete IL-6, and this represents one of the important sources of renal IL-6 [28]. It has also been observed that IL-6 positively promotes immune complex deposition [22].

Recent clinical studies have shown that glomerular endothelial cells are damaged in both acute and chronic IgAN [2]. In a recent study, the amount of proteinuria, hematuria, and glomerular IgA deposition in rats with IgAN was reduced after the infusion of endothelial progenitor cells [29]. Using electron microscopy, in this study, we observed the dissection of glomerular endothelial cells from the basal membrane in rats with IgAN as well as the formation of endothelial cavitation and the insertion of the basal membrane. VE-cadherin, a specific marker of vascular endothelial cells [30] and a component of the adhesion connections between endothelial cells, plays a key role in maintaining vascular integrity and permeability [5]. Our results in this study showed that total VE-cadherin expression in renal tissues was significantly reduced, further confirming the presence of damage to glomerular endothelial cells. Previous studies have shown that IL-6 can reduce the level of Mir-223 in glomerular endothelial cells and lead to cell proliferation, and that ICAM-1 expression and monocyte adhesion can lead to endothelial cell damage [31], also supporting the results of this study.

Additionally, previous studies have shown that the interaction between IL-6 and endothelial cells regulates leukocyte recruitment and inflammatory protein expression [15]. In this study, we investigated the level of VE-cadherin to clarify the effect of IL-6 on HRGEC cell adhesion junctions. The results obtained showed that VE-cadherin protein expression was significantly decreased in HRGECs stimulated using IL-6/IL-6R compared with the unstimulated HRGECs (normal group). Consistent with our findings, a significant decrease in VE-cadherin expression in renal tissue was also reported in a previous study on acute renal injuring with ischemia reperfusion [32]. The downregulation of VE-cadherin can also affect the integrity of tight junctions [33], leading to increased cell permeability. Notably, when VE-cadherin was overexpressed, the increased permeability induced by IL-6/IL-6R was significantly alleviated, suggesting that VE-cadherin was involved in the influence of IL-6/IL-6R on HRGEC permeability. Based on these observations, we hypothesized that a possible mechanism by which IL-6 downregulated VE-cadherin expression is as follows: First, IL-6 and other inflammatory factors may induce VE-cadherin phosphorylation, leading to extracellular exfoliation or VE-cadherin endocytosis [34]. Second, IL-6 may disrupt the VE-cadherin/β-catenin connection, thereby promoting VE-cadherin endocytosis [35], and finally, IL-6 may cause endothelial cells to undergo endothelial mesenchymal transdifferentiation and discard endothelial marker proteins, such as VE-cadherin [36].

However, the permeability of the endothelial cells in the IL-6/IL-6R stimulation group after VE-cadherin overexpression was significantly higher than that corresponding to the cells in the normal VE-cadherin overexpression group. This suggests that IL-6/IL-6R stimulation may have other pathways of action. Reportedly, β-catenin interacts with the cytoplasmic domain of VE-cadherin, helping to increase adhesion strength as well as α-catenin recruitment [37]. In fact, β-catenin is targeted by various tyrosine kinases [38], and its affinity for VE-cadherin is strongly influenced by its phosphorylation status [39]. Notably, β-catenin phosphorylation causes AJ disruption [40], which promotes the dissociation of VE-cadherin/β-catenin [41] by increasing β-catenin phosphorylation at Y654. Furthermore, it has been reported that β-catenin phosphorylation at Y142 reduces the β-catenin/α-catenin association [42]. Our study showed that β-catenin phosphorylation (Y654, Y142) was significantly increased in the IL-6/IL-6R-stimulated HRGECs (Supplementary Figure S3), suggesting that the increased permeability of HRGECs overexpressing VE-cadherin owing to IL-6/IL-6R stimulation may be associated with β-catenin phosphorylation.

The classical pathway of IL-6 is mediated by membrane IL-6R and membrane-bound glycoprotein-130 (gp130) to exert anti-inflammatory effects [43]. Conversely, in the trans-signaling pathway, IL-6 binds to soluble IL-6R to form the IL-6/IL-6R complex, and thereafter binds to gp130 to initiate downstream signals to play a pro-inflammatory role in cells lacking membrane-bound receptors [44]. FITC-dextran permeability analysis showed that IL-6/IL-6R significantly increased the permeability of HRGEC monolayer cells compared with IL-6 (*p* < 0.05). This suggests that IL-6 possibly plays a major role in HRGECs via the trans-signaling pathway. To prove our conjecture, the further silencing of IL-6R blocked the classical pathway, indicating that the VE-cadherin expression downregulation effect of IL-6/IL-6R in HRGEC cells was not alleviated. Conversely, sgp130FC blocked the IL-6 trans-signaling pathway as well as the downregulation of VE-cadherin expression, indicating for the first time that IL-6 primarily affected VE-cadherin expression in HRGEC via the trans-signaling pathway. Studies have also shown that IL-6 leads to the destruction of the human retinal endothelial monolayer barrier, primarily via the trans-signaling pathway [15]. This supports our hypothesis in this study. Soluble IL-6R is formed via protein hydrolysis of membrane-bound receptors, a process known as ectodomain shedding. Alternatively, soluble IL-6R can be secreted directly from cells after selective mRNA splicing [45]. Thus, we speculated that the IL-6-only treatment reduced the TEER values of HRGECs and increased their permeability. This may be because the IL-6R expressed by HRGECs is mainly soluble and mediates the IL-6 trans-signaling pathway. Nevertheless, this requires further study and confirmation.

To the best of our knowledge, this study is the first to show that IL-6 can increase the permeability of HRGEC monolayer cells by decreasing VE-cadherin protein expression through the trans-signaling pathway. However, the study had some limitations. First, even though the rat model of IgAN used in this study is a relatively mature model, the lack of IgA1 in mice may not fully reflect the pathogenesis of IgAN in humans. Second, limited animal experiments were performed in this study, and we also failed to block IL-6 in the IgAN rat model to observe the recovery of endothelial cell injury. Therefore, further clinical studies are required to validate our results.

## Conclusions

In this study, we observed that IL-6 increased the permeability of HRGECs by decreasing VE-cadherin expression via the trans-signaling pathway, providing a theoretical basis for understanding the mechanism of IgAN glomerular endothelial cell injury.

## Supplementary Information

Below is the link to the electronic supplementary material.

Supplementary file1 (DOC 17 KB)

Supplementary file2 (DOC 519 KB)

Supplementary file3 (DOC 685 KB)

## Data Availability

Data that support the results of the present study are available from the corresponding author upon reasonable request.

## Abbreviations

IgAN: IgA nephropathy
IL-6: Interleukin 6
HRGECs: Human renal glomerular endothelial cells
TEER: Trans-endothelial resistance
VE-cadherin: Vascular endothelial-cadherin
AJs: Adherens junctions
Gd-IgA1: Galactose deficient IgA1
SPF: Specific pathogen-free
BSA: Bovine serum albumin
CCl4: Carbon tetrachloride
LPS: Lipopolysaccharide
IF: Immunofluorescence
Wb: Western blotting
RBC: Red blood cell
FBS: Foetal Bovine Serum
PBS: Phosphate buffered saline
FITC: Fluorescein isothiocyanate
GAPDH: Glyceraldehyde phosphate dehydrogenase
PAS: Periodic acid-Schiff
ELISA: Enzyme-linked immunosorbent assay
ANOVA: One-way analysis of variance
DAPI: 4′, 6-Diamidino-2-phenylindole
CCK-8: Cell Counting Kit-8
SD: Standard deviation
IL-6R: IL-6 receptor
gp130: Glycoprotein-130
