# Supplementary material for: Interleukin-6 Downregulates the Expression of Vascular Endothelial-Cadherin and Increases Permeability in Renal Glomerular Endothelial Cells via the Trans-Signaling Pathway
Source: Inflammation. 2022 Jul 23;45(6):2544–58. doi: 10.1007/s10753-022-01711-3 (PMC9646551; doi:10.1007/s10753-022-01711-3)
Supplement: Supplementary file 1 — Supplementary file1 (DOC 17 KB) [file 10753_2022_1711_MOESM1_ESM.doc]

Table S1. Primer sequences of human genes for qRT‑PCR

| **Primer name** | **Primer sequence (5’-3’)** |
| --- | --- |
| IL-6R (Forward) | GAGGAGTTCGGGCAAGG |
| IL-6R (Reverse) | CCAGCAACCAGGAATGTG |
| VE-cadherin (Forward) | CCTACCAGCCCAAAGTG |
| VE-cadherin (Reverse) | GTTATCGTGATTATCCGTGA |
| β-actin (Forward) | CACTGTGCCCATCTACGAGG |
| β-actin (Reverse) | TAATGTCACGCACGATTTCC |
