# Supplementary material for: Interleukin-6 Downregulates the Expression of Vascular Endothelial-Cadherin and Increases Permeability in Renal Glomerular Endothelial Cells via the Trans-Signaling Pathway
Source: Inflammation. 2022 Jul 23;45(6):2544–58. doi: 10.1007/s10753-022-01711-3 (PMC9646551; doi:10.1007/s10753-022-01711-3)
Supplement: Supplementary file 2 — Supplementary file2 (DOC 519 KB) [file 10753_2022_1711_MOESM2_ESM.doc]

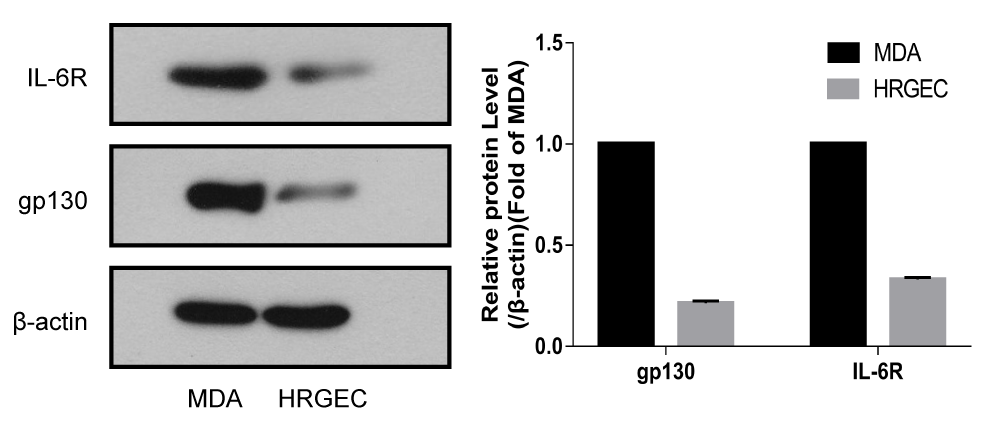


Supplementary Figure S2. Expression of IL-6R and gp130 in HRGEC. gp130 and IL-6R were expressed in HRGEC, but their expression levels were lower than MDA cells (p <0.01).

Abbreviations: HRGEC, human renal glomerular endothelial cell; MDA, Breast cancer cell MDA-MB-231; IL-6R, interleukin-6 receptor; gp130, glycoprotein 130.
