# Supplementary material for: Interleukin-6 Downregulates the Expression of Vascular Endothelial-Cadherin and Increases Permeability in Renal Glomerular Endothelial Cells via the Trans-Signaling Pathway
Source: Inflammation. 2022 Jul 23;45(6):2544–58. doi: 10.1007/s10753-022-01711-3 (PMC9646551; doi:10.1007/s10753-022-01711-3)
Supplement: Supplementary file 3 — Supplementary file3 (DOC 685 KB) [file 10753_2022_1711_MOESM3_ESM.doc]

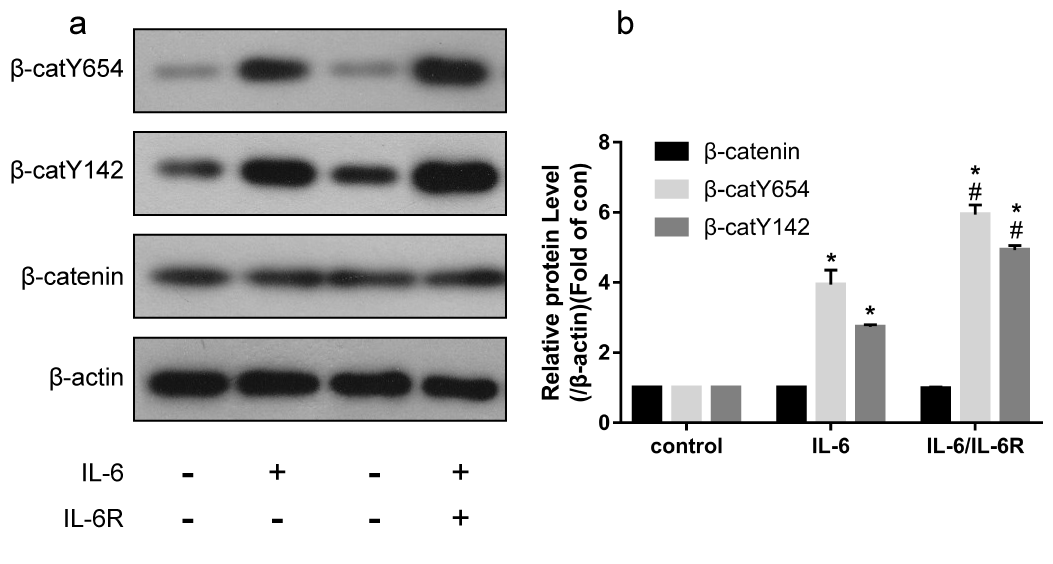


Supplementary Figure S3. **Phosphorylation of β-catenin in different HRGEC groups.** (a) Western blotting showed that IL-6 or IL-6/IL-6R significantly increased the phosphorylation of β-catenin at Y142 and Y654. (b)Statistical analysis showed that IL-6 /IL-6R increased beta-catenin phosphorylation significantly more than IL-6 alone (P < 0.01). *p < 0.01, compared with control; #p < 0.01, compared with IL-6 group.

Abbreviations: HRGEC, human renal glomerular endothelial cell; IL-6, interleukin-6; IL-6R, interleukin-6 receptor; β-cat, β-catenin.
